# Retrogradation Regulates the Thermal Puffing Quality of Tigernut (*Cyperus esculentus* L.) Starch via Modulating Its Multi-Scale Structure and Water State

**DOI:** 10.3390/foods15203612

**Published:** 2026-10-11

**Authors:** Yuchen Wu, Siqi Wu, Sevenur Sarıkaya, Heba G. R. Younis, Qihui Mao, Fayin Ye

**Affiliations:** 1 China-Hungary Belt and Road Joint Laboratory on Food Science, College of Food Science, Southwest University, Chongqing 400715, China; 15978589931@163.com (Y.W.); wsq_0929@163.com (S.W.); sevenur.98@gmail.com (S.S.); maoqihui2022@126.com (Q.M.); 2 Agricultural Engineering Department, Faculty of Agriculture, Cairo University, Giza 12613, Egypt; hebagamal73@agr.cu.edu.eg; 3 Chongqing Sweet Potato Engineering and Technology Research Center, Chongqing 400715, China; 4 Chongqing Key Laboratory of Speciality Food Co-Built by Sichuan and Chongqing, Chongqing 400715, China

**Keywords:** tigernut starch, retrogradation, thermal puffing, water distribution, multi-scale structure, correlation analysis

## Abstract

Starch retrogradation is a key process governing the final quality of indirectly thermally puffed starchy foods. This study investigated the effect of retrogradation duration (0–72 h) on the multi-scale structure, water distribution, thermal puffing performance and textural properties of tigernut starch-based products. XRD, FTIR and synchrotron SAXS analyses confirmed that retrogradation progressively promoted molecular ordering, as reflected by reduced lamellar long period (*d*_Bragg_) and radius of gyration (*R*_g_), along with enhanced short-range order (*R*_1047/1022_) and relative crystallinity. LF-NMR results showed that among all tested retrogradation durations, the 4 h treatment presented the highest proportion of immobilized water (*A*_22_), while longer retrogradation periods led to a gradual increase in the content of free water. Puffing performance and texture showed a non-linear dependence on retrogradation time: the 4 h retrograded sample presented the best comprehensive quality among the seven tested time points, with the highest expansion ratio (9.75), specific volume (8.42 mL/g) and crispness (36.67), and the lowest hardness (1165.53 g). Pearson correlation analysis identified water activity (*a*_w_) as the dominant factor governing puffing performance, which was in turn regulated by retrogradation-induced structural evolution. This study elucidates that a moderate retrogradation degree (approximately 4 h) facilitates the formation of a moderately ordered lamellar structure with the most favorable water retention performance within the current experimental range, creating ideal thermodynamic conditions for puffing, while excessive retrogradation causes structural rigidity and poor expansion.

## 1. Introduction

Tigernut (*Cyperus esculentus* L.), an underutilized crop with significant ecological and economic value, has recently gained recognition as a novel food ingredient [1]. Rich in lipids, proteins, carbohydrates (specifically starch accounting for 20–25% on a dry weight basis), and bioactive compounds (such as alkaloids, phytates, flavonoids, etc.), tigernuts offer high potential for food and pharmaceutical applications [2]. While traditionally consumed directly or processed into beverages (like “horchata”) and oils [3], recent interest has focused on utilizing tigernut flour and starch as functional ingredients in noodles, pasta, baked goods and starch-based puffed snacks [4,5].

Indirect thermal puffing is a typical high-temperature short-time processing technology to produce porous, crispy, and low-density snack foods [6,7]. This process generally relies on the rapid vaporization of internal moisture to expand the starch matrix [7]. Its final puffing performance is jointly determined by two core factors: the elastic deformability of the pre-formed starch gel network, and the existing state and distribution of internal water populations [6,8]. While extrusion puffing is common, indirect thermal puffing (such as deep frying or baking pre-formed gels) allows for precise control over the final product’s texture. The quality of puffed starch-based foods, specifically expansion ratio and crispness, is heavily dependent on the physicochemical properties of the starch matrix prior to puffing [9,10].

Retrogradation, a process involving the re-association of starch molecules after gelatinization into ordered structures during cooling and storage, is a crucial yet complex factor influencing the techno-functional properties of starch, including its thermal puffing capacity. Jiamjariyatam et al. [11] reported that, after retrogradation at 4 °C for 24 h, rice starch gel formed a more continuous network structure with the increasing amylose content, which led to puffed products with finer and more evenly distributed air cells. Extended retrogradation time increased the relative crystallinity of dried rice starch gel pellets, and higher relative crystallinity led to smaller, denser air cell structures and reduced expansion, which further enhanced the crispness, hardness and fracturability of the final puffed products [12]. Conversely, excessive retrogradation under prolonged storage can lead to an overly rigid, highly crystalline structure that cannot undergo sufficient elastic deformation during high-temperature pressurization, thus failing to expand effectively [13]. Water in pre-formed starch gels acts as both a plasticizer and a blowing agent during thermal puffing, making its state a critical determinant of the final product’s microstructure and texture. Retrogradation induces changes in starch gels at multiple structural levels, from double-helix formation to crystalline perfection, which inevitably alter the water binding capacity and distribution within the starch matrix. Controlled retrogradation as a pre-treatment for indirect thermal puffing can modify the multi-scale structure and water state of pre-formed starch gels in a progressive manner. By precisely regulating the retrogradation duration, it is feasible to tailor the degree of molecular ordering, adjust the proportion of immobilized water in the matrix, and avoid the excessive structural consolidation caused by long-term amylopectin recrystallization. In this work, we define “moderate retrogradation” explicitly as the specific intermediate stage that the short-term ordered rearrangement of leached amylose is substantially completed, while extensive recrystallization of the outer branches of amylopectin has not yet initiated. This pre-regulation on multi-scale structure and water distribution further creates the most favorable internal vapor pressure condition during thermal puffing, and accordingly delivers improved expansion performance compared with non-retrograded and excessively retrograded samples [14].

Tigernut starch, with its distinct granule size distribution and characteristic chemical composition, has attracted growing attention as a promising novel ingredient for expanded healthy snack products [1,2]. Compared with widely studied rice, corn and potato starches, it exhibits unique inherent properties including a typical A-type crystalline pattern, granule size ranging from 2 to 18.53 μm, and an amylose content varying between 9.71% and 27.01% [15]. These features make its retrogradation pathway and water migration behavior substantially different from those of conventional starch systems. However, the puffing behavior of tigernut starch and the regulatory role of controlled retrogradation in this process still remain poorly documented. Most previous studies on starch-based puffing systems focused on native or fully gelatinized materials, while the structural and physicochemical transformations occurring during puffing have been well described for common starches, the specific impact of retrogradation on the multi-scale structure of tigernut starch (covering molecular order, crystal growth and lamellar arrangement), water population dynamics, and their combined influence on thermal puffing performance has not been fully elucidated. To date, a clear mechanistic connection linking retrogradation-induced multi-scale structural evolution, water redistribution, and final puffing functionality is still not fully established.

Therefore, this study aims to: (1) characterize the multi-scale structural evolution (long-range crystalline, short-range, and lamellar) of tigernut starch gel during retrogradation (0–72 h) in 4 °C refrigerator using XRD, FTIR, and SAXS; (2) monitor the corresponding changes in water distribution and state using LF-NMR and water activity analysis; (3) evaluate the resulting thermal puffing properties (expansion ratio, specific volume) and textural attributes (hardness, crispness) of the puffed products; and (4) establish the correlation between structural parameters, water state, and puffing quality to unravel the underlying regulatory mechanism. This work provides fundamental insights and a practical processing window for optimizing the quality of tigernut starch-based puffed foods.

## 2. Materials and Methods

### 2.1. Materials

Tigernut, produced in Longhui County, Hunan Province, China, was harvested in September 2023 and purchased in a dried state after post-harvest sun-drying. Rapeseed oil (food grade) was purchased from COFCO International (Beijing) Co., Ltd. (Beijing, China). α-amylase (S31302-50ku, from porcine pancreas, ≥5 u/mg solid) was purchased from Shanghai Yuanye Bio-Technology Co., Ltd. (Shanghai, China). All other chemicals and reagents were of analytical grade.

### 2.2. Extraction of Tigernut Starch

The dried tigernuts were soaked in deionized water overnight (12 h), and the excess surface water was drained. The mixture was then blended in a grinder at a material-to-water mass ratio of 1:4 for 3 min. The slurry was repeatedly filtered through a 100-mesh sieve to obtain the filtrate, and the residue was ground again, with the filtrate also passed through a 100-mesh sieve. The two filtrates were combined and allowed to settle. The precipitate was repeatedly washed with deionized water and centrifuged (1789× *g*, 20 min) to remove the upper oil layer and the brownish substance on the starch surface until the precipitate turned pure white. The precipitate was collected and dried in an oven at 40 °C, then ground and sieved through a 100-mesh sieve to obtain tigernut starch. The tigernut starch samples were sealed in airtight plastic bags and stored in a desiccator for subsequent experiments.

### 2.3. Preparation of Retrograded Tigernut Starch

The tigernut starch and deionized water were weighed in a 1:1.5 (*w*/*w*) ratio, stirred evenly, poured into an aluminum tray, and placed in a steaming pot (with an inner diameter of 28 cm; Yonghui Superstores Co., Ltd., Fuzhou, China) for 2.5 min at 2200 W. The pre-gelatinized samples were then retrograded in a 4 °C refrigerator for 0 h, 4 h, 8 h, 16 h, 24 h, 48 h, and 72 h. After retrogradation, the retrograded samples were shaped using a 2 cm circular mold and dried in an electric oven (DHG-9140, Shanghai Qixin Scientific Instrument Co., Ltd., Shanghai, China) at 65 °C until the moisture content reached approximately 10%. Samples with different retrogradation durations (0–72 h) were designated as TS-X, where X represented the corresponding retrogradation hour. For the analyses of degree of retrogradation (DR), X-ray diffraction (XRD), Fourier transform infrared spectroscopy (FTIR) and small-angle X-ray scattering (SAXS), a portion of the dried starch gel was crushed using a grinder (SHM2002, Zhejiang Shaoxing Supor Household Appliances Co., Ltd., Shaoxing, China), sieved through a 100-mesh sieve. After moisture equilibration, the powdered samples were sealed in airtight plastic bags and placed in a desiccator. The amylose content was determined using the spectrophotometric method described in GB/T 15683-2025, Inspection of grain and oils—Determination of amylose content in rice, with a method validation extension adapted for tigernut starch [16]. The amylose content of the isolated tigernut starch was determined to be 20.55% (dry basis).

### 2.4. Determination of the Degree of Retrogradation

The degree of retrogradation (DR) was measured according to the method of Tsuge et al. [17] with slight modifications. 250 mg of the powdered sample was homogenized with 50 mL of distilled water for 3 min. A 5 mL of the homogenized solution was mixed thoroughly with 3 mL of distilled water, 2 mL of 0.1 M phosphate buffer (pH 6.0), and 2 mL of diluted α-amylase solution (3.5 units/mL). After incubating the mixed solution at 37 °C for 10 min, 5 mL of 4 mol/L NaOH was added to terminate the enzymatic reaction. The solution pH was adjusted to 7.0 with 4 mol/L HCl and then diluted to 100 mL with distilled water. A 10 mL portion of the diluted solution was collected, added to 5 mL of 0.2% I_2_–2% KI solution (*w*/*w*) in a volumetric flask, and diluted to 100 mL with distilled water. After incubating at 25 °C for 15 min, the absorbance was measured at 625 nm using a UV spectrophotometer (T6, Beijing Purkinje General Instrument Co., Ltd., Beijing, China). The degree of retrogradation (DR) was calculated according to Equation (1):

DR (%) = (*b* − *c*)/(*a* − *c*) × 100 (1)

where *a*—absorbance of total starch, measured without α-amylase, with all other steps identical to the above method; *b*—absorbance of starch after enzymatic reaction of the sample under the above conditions; *c*—absorbance of residues after complete starch digestion, blank control to eliminate non-starch matrix interference.

### 2.5. X-Ray Diffraction (XRD)

The crystalline structure of the tigernut starch in dried gels was analyzed using an X-ray diffractometer (X’Pert^3^ Powder10300, Malvern Panalytical Ltd., Almelo, The Netherlands). The powdered sample was placed at room temperature for 24 h to balance the moisture and eliminate its impact on the test. XRD patterns were recorded at 40 kV and 40 mA with Cu-Kα radiation (*λ* = 0.154 nm). The diffraction angle was measured in the range of 4–40° (2*θ*) with a scan speed 4°/min, divergence slit width: 1° and scan step size 0.01°. The relative crystallinity (RC) was analyzed by MDI Jade 6.5 software (Materials Data Inc., Livermore, CA, USA) using the method described by [18].

### 2.6. Fourier Transform Infrared Spectroscopy (FTIR)

The infrared spectra of the powdered samples were recorded on an attenuated total reflectance Fourier transform infrared (ATR-FTIR) spectrometer (Spectrum 100, PerkinElmer, Waltham, MA, USA) equipped with a diamond ATR crystal. The spectra were collected in the wavenumber range of 600–4000 cm^−1^ with a resolution of 4 cm^−1^ and the scan time of 32. The instrument’s built-in software was used to smooth the curve, adjust the data, correct the baseline, and normalize the spectrum. The obtained spectrum was then subjected to Fourier deconvolution using OMNIC software (Version 9.2, Thermo Fisher Scientific, Madison, WI, USA), with a peak width of 40 cm^−1^ and an enhancement factor of 1.9. The band intensity values of the characteristic peaks near 1047 cm^−1^, 1022 cm^−1^, and 995 cm^−1^ were recorded. The band intensity ratios of *R*_1047/1022_ and *R*_995/1022_ were calculated to analyze the short-range ordered structure [19].

### 2.7. Small-Angle X-Ray Scattering (SAXS)

SAXS experiments were carried out on the BL16B1 beamline equipped with a Pilatus 2MF detector at the Shanghai Synchrotron Radiation Facility (Shanghai Advanced Research Institute, Shanghai, China). Silver behenate was used as the calibration standard for the SAXS scattering vector. Prior to measurement, 0.2 g of the powdered sample was mixed with distilled water at room temperature to make a suspension (25%, *w*/*v*). The suspension was transferred to the sample cell and exposed to a synchrotron beamline with a photon energy of 10 keV (corresponding to a wavelength of 0.124 nm) for 40 s, with a sample-to-detector distance of 1900 mm, and the beam current of 180.38 mA. The scattering vector (*q* = 4π sin*θ*/*λ*, where 2*θ* was the scattering angle) was in the range of 0.15 < *q* < 1.5 nm^−1^, and scattering intensities were recorded using a photodiode to obtain SAXS data. The two-dimensional scattering patterns were converted into one-dimensional data using FIT2D.exe software (Version 10.132, European Synchrotron Radiation Facility, Grenoble, France), followed by background subtraction and normalization for all datasets, from which parameters such as the radius of gyration (*R*_g_), fractal dimension (*D*_m_), and lamellar long period (*d*_Bragg_) were calculated by the method of [20].

### 2.8. Determination of Moisture Content and Water Activity

The moisture content was determined using the direct drying method specified in GB 5009.3-2016, National Food Safety Standard—Determination of Moisture in Foods [21]. The water activity (*a*_w_) was measured using a water activity meter (HD-6A, Wuxi Huake Instrument Co. Ltd., Wuxi, China). After moisture equilibration, the powdered sample (obtained in Section 2.3) was carefully transferred into a standard water activity sample cup for measurement. The sample cup containing the test substance was then placed into the sensor base of the water activity meter and kept in a sealed state. The measurement was performed by the launched “Smart Water Activity Meter” application.

### 2.9. Determination of Water Distribution

The water distribution in the dried tigernut starch gel was determined using a low-field nuclear magnetic resonance (NMI20-060V-I, Suzhou Niumag Analytical Instrument Co., Ltd., Suzhou, China) based on transverse relaxation time (*T*_2_). The dried retrograded tigernut starch gel prepared in Section 2.3 was placed into a 25 mm NMR tube, and analyzed for *T*_2_ relaxation using the Carr-Purcell-Meiboom-Gill (CPMG) mode. The operating parameters were as the following: the spectral frequency (SF) was set at 20 MHz, with a 90° pulse time (P1) of 7.52 μs and a 180° pulse time (P2) of 15.52 μs. The detection parameters were set as follows: signal sampling points (TD) = 26,686, sampling bandwidth = 333.33 KHz, waiting time (TW) = 6000 ms, number of signal averages (NS) = 8, echo time (TE) = 0.1 ms, and number of echoes (NECH) = 8000. The transverse relaxation time (*T*_2_) and peak area (*A*_2_) at different relaxation times were obtained by analyzing the resulting curve [22].

### 2.10. Preparation of Puffed Tigernut Starch

The sample of dried retrograded tigernut starch gels was puffed by a deep-frying method. The rapeseed oil in the deep-fried pan was preheated to around 190 °C on an induction cooker (Model: CH21203D; Guangdong Galanz Life Electrical Appliances Manufacturing Co., Ltd., Zhongshan, China). Then, the sample was immersed in the rapeseed oil for deep-frying for 10 s. After oil drainage and cooling to room temperature, and wiping off the excess oil from the surface of the puffed sample with paper towels, the puffed samples was put in a plastic bag, sealed and stored at room temperature for subsequent analysis.

### 2.11. Determination of Expansion Ratio and Specific Volume of Puffed Samples

The volume of the gel samples before and after thermal expansion was measured by the method of [23]. Firstly, the sample to be tested was weighed and recorded as *m*_1_. Then, the volume of the sample was determined by rapeseed displacement, with the reading taken from a graduated cylinder as *V*_1_. The sample was then deep-fried for expansion, as described in Section 2.10. The mass of puffed sample (*m*_2_) and its volume (*V*_2_) were measured using the same rapeseed displacement method. The expansion ratio and specific volume of the puffed samples were calculated according to Equations (2) and (3), respectively.

Expansion ratio = *V*_2_/*V*_1_ (2)

Specific volume (mL/g) = *V*_2_/*m*_2_ (3)

### 2.12. Texture Profile Analysis (TPA)

The hardness and crispness of the puffed samples was carried out using a texture analyzer (TA. XT plus, Stable Micro Systems, Godalming, Surrey, UK) by the method of [24] with slight modification. The measurement was performed using a P/0.5s probe with the following parameters: pre-test speed 2.0 mm/s, test speed 1.0 mm/s, and post-test speed 2.0 mm/s; compression depth, 3 mm; trigger force, 10.0 g. The peak maximum of the first compression curve was defined as hardness [25], while crispness was quantified as the number of fracture peaks in the first-compression force-displacement curve [26].

### 2.13. Statistical Analysis

All independent experiments were performed at least in triplicate, and the data were expressed as mean ± standard deviation. Statistical analysis was conducted using SPSS 27.0 (IBM Corp., Armonk, NY, USA) with one-way analysis of variance (ANOVA), and the comparisons between the data were performed using Duncan’s multiple range test with a significance level of *p* < 0.05. The correlation analysis was conducted using the Pearson method in the Correlation Plot of Origin 2021 (OriginLab Corp., Northampton, MA, USA).

## 3. Results

### 3.1. Retrogradation Degree of Dried Tigernut Starch Gel

**Figure 1:**
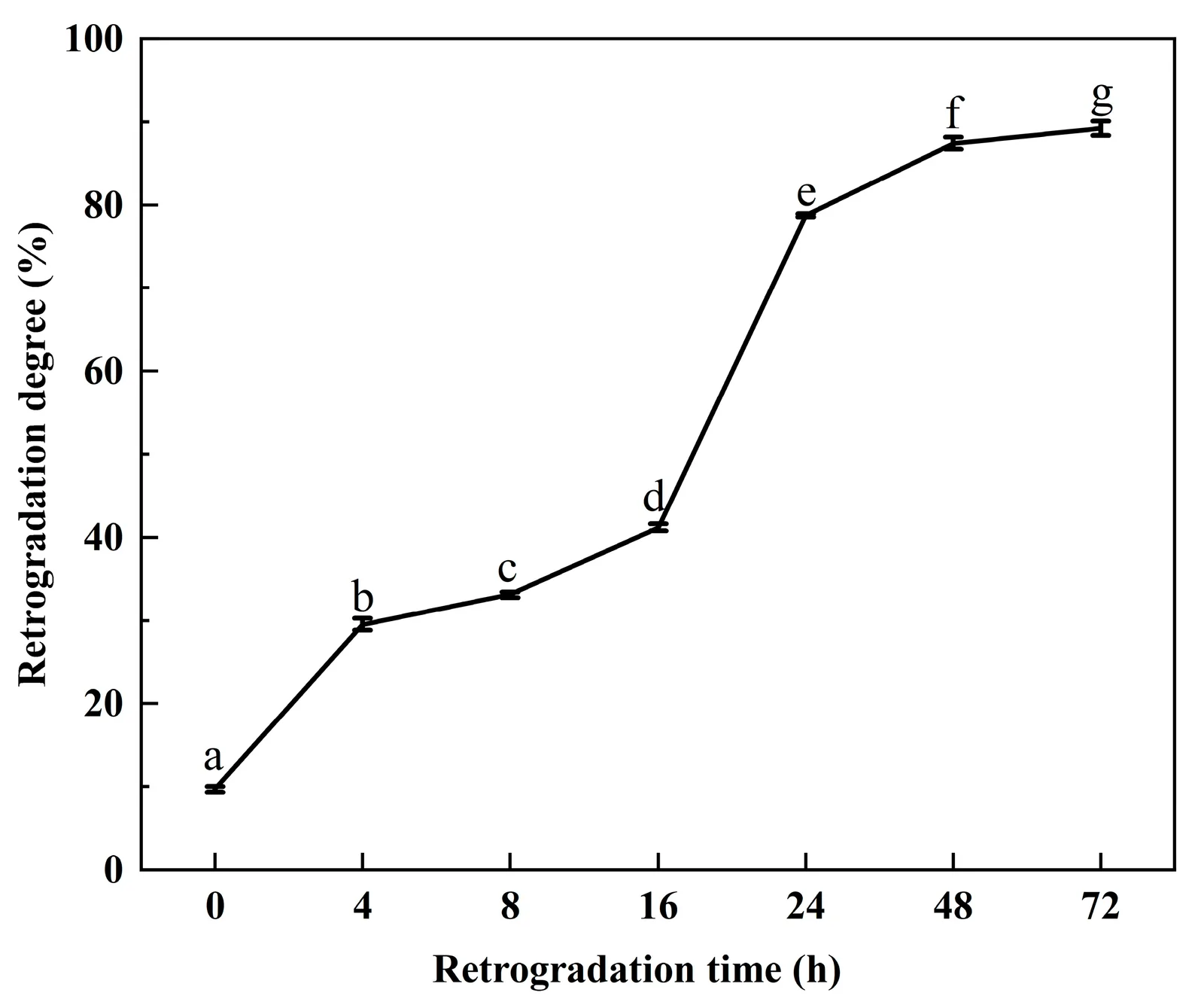
Changes in retrogradation degree of tigernut starch gels during low-temperature storage (4 °C, 0–72 h) and subsequent oven drying. Different lowercase letters above the line chart indicate significant differences (*p* < 0.05).

The degree of retrogradation is an index to quantify the realignment and recrystallization of gelatinized starch molecules during cooling and storage. Widely adopted qualitative and quantitative methods for retrogradation degree determination include enzymatic assays, mechanical property tests (e.g., texture profile analysis, rheological measurement), spectroscopic analysis (e.g., X-ray diffraction, Fourier-transform infrared spectroscopy) [27], and thermal analysis (e.g., differential scanning calorimetry) [28]. The enzymatic method is based on the principle that native starch and retrograded starch generally exhibit significantly lower accessibility to α-amylase than gelatinized starch, showing a much slower and limited hydrolysis rate under the same experimental conditions [17]. The *α*-amylase preferentially cleaves α-1,4-glycosidic bonds in gelatinized and amorphous starch, and the remaining resistant residue is quantified to determine the molecular ordered conformation of retrograded starch. Figure 1 shows the retrogradation degree of tigernut starch gel determined via the α-amylase-iodine method at different retrogradation durations. As retrogradation time extended, the retrogradation degree of tigernut starch increased significantly from 9.69% to 89.17% (*p* < 0.05). The retrogradation degree rose rapidly within the first 24 h, while the growth rate gradually slowed down and reached a plateau thereafter. Starch retrogradation consists of two sequential stages: the short-term rapid retrogradation dominated by amylose double-helix formation, and the long-term slow rearrangement of amylopectin molecules. After 24 h of storage, amylopectin recrystallization dominated the retrogradation process. Subsequent sections will analyze the multi-scale structural evolution and thermal expansion properties of tigernut starch with different retrogradation degrees.

### 3.2. Multi-Scale Structural Evolution of Tigernut Starch During Retrogradation

The structural evolution of tigernut starch gels at different retrogradation durations was systematically investigated using X-ray diffraction (XRD), Fourier transform infrared spectroscopy (FTIR), and synchrotron radiation small-angle X-ray scattering (SAXS).

#### 3.2.1. Long-Range Crystalline Structure

**Figure 2:**
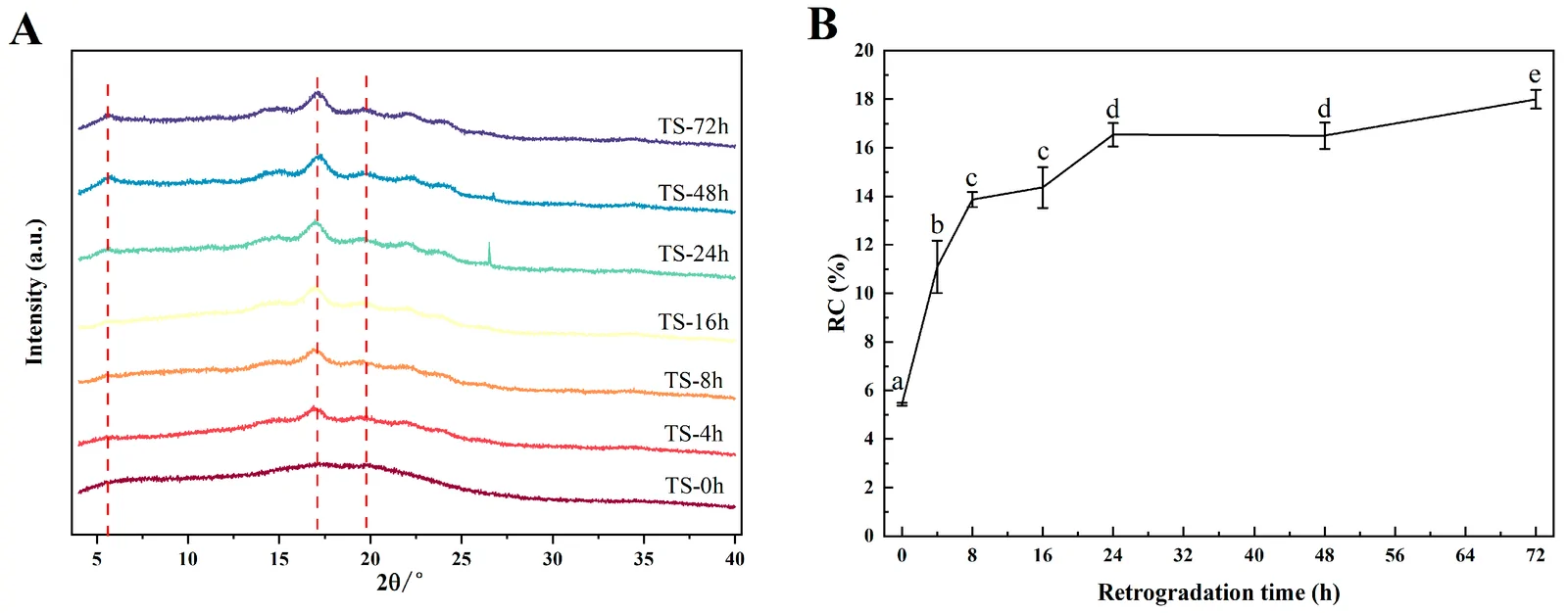
X-ray diffraction patterns (**A**) and relative crystallinity (RC) (**B**) of dried tigernut starch gels as a function of retrogradation time (0–72 h). TS-suffix hour numbers in the figure represent the sample codes corresponding to different retrogradation durations. Different lowercase letters above the line chart indicate significant differences (*p* < 0.05).

As shown in Figure 2A, the X-ray diffraction curve of the 0 h retrogradation sample exhibited no distinct diffraction peaks, which was due to the disruption of starch molecular ordered structures during gelatinization. As retrogradation time extended from 4 h to 72 h, distinct crystalline peaks appear at approximately 5.6°, 15°, 17°, 20°, and 22° (2*θ*) in the diffraction patterns. The characteristic diffraction peaks of tigernut starch became more pronounced, and the corresponding peak area gradually increased, indicating the continuous formation and perfection of ordered double-helical structures in starch molecules during retrogradation. Notably, a well-resolved peak at 2*θ* = 17° was observed in all retrograded samples (Figure 2A), which was primarily assigned to the recrystallization of amylopectin double helices [29]. A distinct new characteristic peak was observed at 2*θ* ≈ 20°, corresponding to the typical V-type crystalline structure, which is widely recognized to originate from the formation of amylose inclusion complexes with small guest molecules [30]. An et al. [29] reported that wheat starch gels underwent continuous retrogradation during 4 °C refrigerated storage, accompanied by an increasing number of distinct crystalline peaks, elevated relative crystallinity, and a gradual reduction in amorphous regions. By curve fitting and integration, the relative crystallinity (RC) of samples at different retrogradation durations was calculated and summarized in Table 1. The RC of the dried starch gels showed an overall upward trend, significantly increasing from 5.44% at 0 h to 17.99% at 72 h (*p* < 0.05) (Table 1). Long-range order characterizes the extent of intermolecular interaction and rearrangement between amylose and amylopectin, which drives the formation of new ordered crystalline structures during retrogradation. As shown in Figure 2B, the growth of RC followed a two-stage pattern: the RC increases rapidly within 0 h to 8 h, followed by a significantly slower growth phase (8–72 h). Jiamjariyatam et al. [11] reported that in rice starch gels subjected to 72 h retrogradation, the RC was significantly governed by amylose content and storage duration. The waxy rice starch sample with 0.1% amylose content showed no detectable retrogradation signals within 24–48 h. In starch gels with amylose content above 4.00%, amylose rapidly re-associated within hours to form seed nuclei that subsequently promote amylopectin recrystallization after 24 h of storage. Consistent with this mechanism, the 0–8 h retrogradation stage in the present study corresponds to the rapid completion of amylose recrystallization, followed by the recrystallization of amylopectin outer chains under prolonged storage. Similarly, Chen et al. [31] found that when cooked potato tubers were stored at 4 °C, the relative crystallinity reached 20.55% for 1-day storage, and remained at 22.59% and 22.47% for 3-day and 7-day storage, respectively.

#### 3.2.2. Short-Range Order Structure

**Figure 3:**
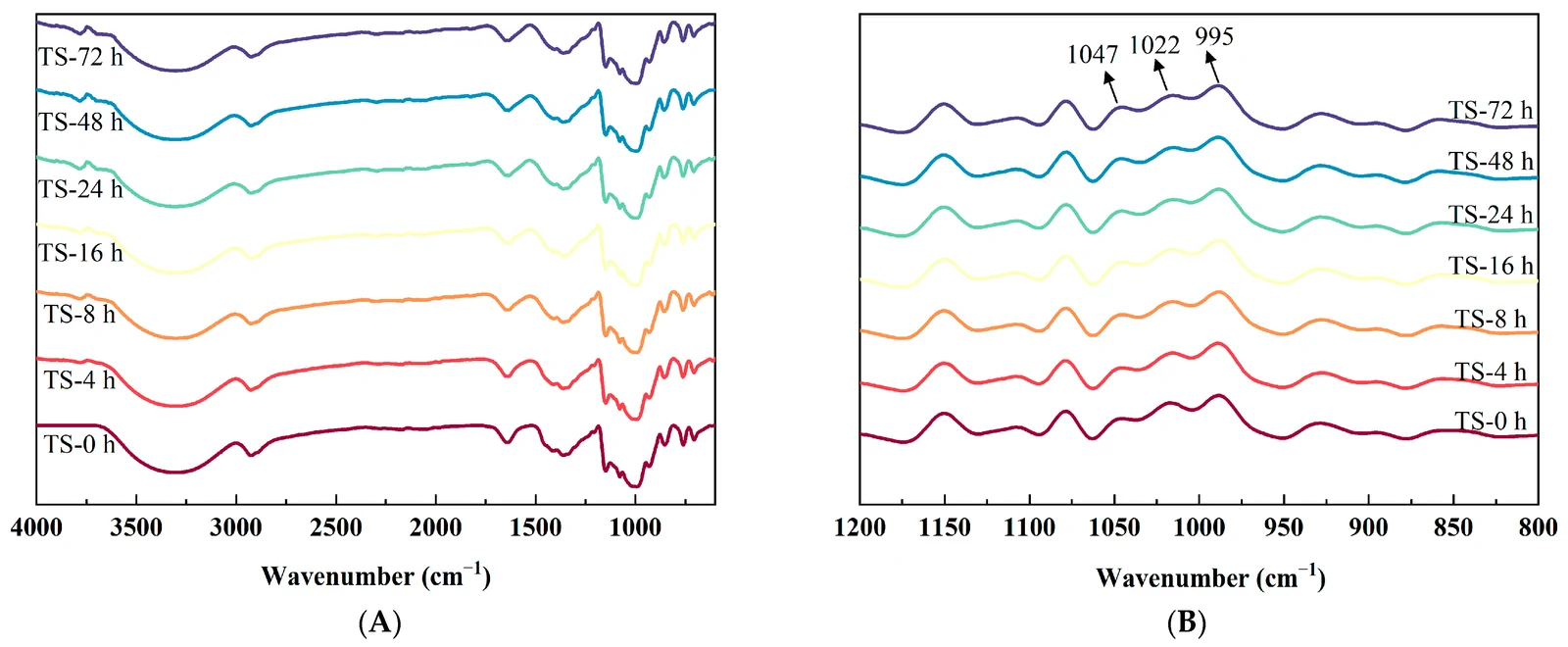
Infrared spectra (**A**) and Fourier deconvoluted FTIR spectra (**B**) of dried tigernut starch gels as a function of retrogradation time (0–72 h). TS-suffix hour numbers in the figure represent the sample codes corresponding to different retrogradation durations.

The FT-IR spectra (Figure 3A) indicate that the positions of the main characteristic bands of starch do not change significantly after retrogradation and drying, suggesting that these two treatments do not alter the covalent chemical bonds or inherent functional groups in starch molecules. Meanwhile, a new characteristic absorption band appears at 3750 cm^−1^ in the dried retrograded starch gels and the intensity of this band gradually increases with the extension of retrogradation time. This peak is assigned to the stretching vibration of free hydroxyl (O-H) that does not form intermolecular or intramolecular hydrogen bonds [32,33]. The generation of such free hydroxyl groups originates from the breakage of partial associated hydrogen bonds during the rearrangement of hydrogen bond networks in starch retrogradation. Furthermore, the 1200–800 cm^−1^ region is the characteristic fingerprint region corresponding to the short-range ordered structure of starch molecules [33]. Fourier self-deconvolution is performed on the original spectra within this range (Figure 3B), and the derived parameters are summarized in Table 1. The *R*_1047/1022_ absorbance ratio, calculated from the normalized FT-IR spectra, reflects the short-range order degree of starch molecular chains. The results show that as retrogradation time extends, *R*_1047/1022_ gradually increases from 0.5086 to 0.6039, indicating a progressive enhancement in the short-range ordered structure of starch molecules [34]. The *R*_995/1022_ ratio represents the double helicity of starch molecules, which characterizes the packing state of double helices in crystalline regions. Compared with the 0 h retrogradation sample, *R*_995/1022_ shows an overall increasing trend. As summarized in Table 1, the long-range order (relative crystallinity) and short-range order (*R*_1047/1022_) of tigernut starch exhibit consistent variation trends with increasing retrogradation time. The parallel increase in the two indicators demonstrates that retrogradation promotes the formation of double helices and crystalline structures, which drives the ordered rearrangement of starch molecules and enhances the overall internal structural order.

#### 3.2.3. Lamellae Structure

**Figure 4:**
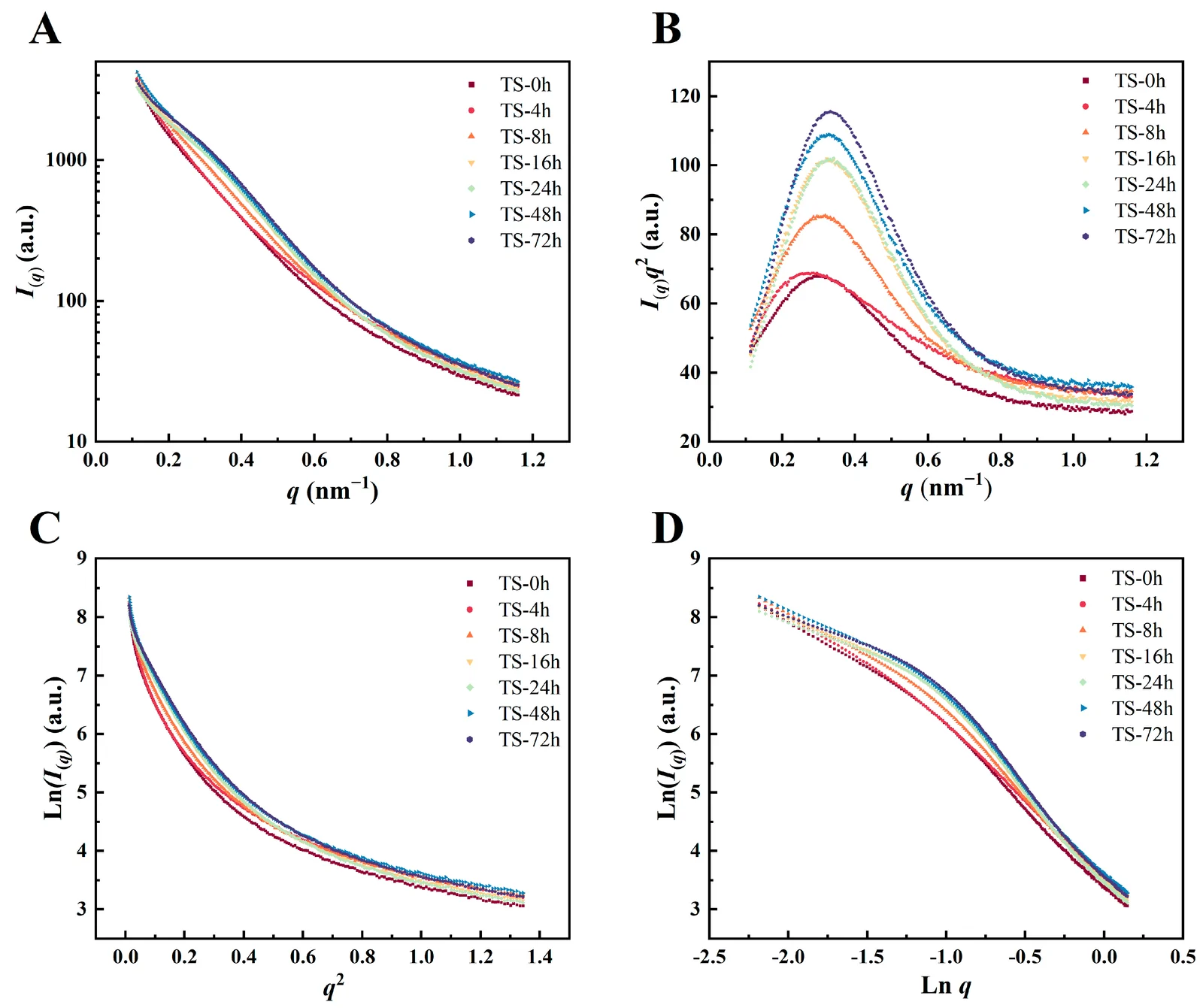
Small-angle X-ray scattering (SAXS) curves of dried tigernut starch gels as a function of retrogradation time (0–72 h). (**A**) 1D SAXS patterns (*I*_(q)_ versus *q*); (**B**) A Lorentz-corrected 1D plots (*I*_(q)_ × *q*^2^ versus *q*); (**C**) Guinier plots (*I*_(q)_ versus *q*^2^); (**D**) Double-logarithmic plots (ln *I*_(q)_ versus ln *q*).

Small-angle X-ray scattering (SAXS), as an analytical technique capable of characterizing material structural features within the scattering vector range of 0.001 < *q* < 5 nm^−1^, is widely applied in the study of starch lamellar structures [30]. Figure 4A presents the one-dimensional SAXS scattering profile of scattering vector *q* versus scattering intensity *I*_(*q*)_. For native starch materials, this curve typically exhibits a distinct characteristic scattering peak near *q* = 0.6 nm^−1^, which corresponds to the ~10 nm lamellar long period originating from the alternating arrangement of crystalline and amorphous regions in starch semi-crystalline structure. In the present study, the tigernut starch is gelatinized to disrupt the native granular and crystalline lamellar structure; consequently, no well-defined characteristic scattering peak corresponding to native crystalline lamellae is observed in all retrograded starch gel samples. However, the scattering intensity, which is directly correlated with the electron density contrast between crystalline and amorphous domains, shows significant differences among groups. Compared with the 0 h retrogradation sample, all retrograded samples exhibit higher scattering intensity, and the scattering intensity gradually increases with the extension of retrogradation time. This trend indicates that retrogradation promotes the rearrangement of starch molecules into ordered double-helical structures, which enhances the overall molecular order and improves the orientation uniformity of double helices within the reorganized semi-crystalline lamellae.

The one-dimensional scattering profile in Figure 4A is processed via Lorentz correction to obtain the *I*_(*q*)_*q*^2^ ~ *q* curve (Figure 4B) [20]. All samples exhibit a broad Bragg peak near *q* = 0.3 nm^−1^, which corresponds to the newly reorganized semi-crystalline lamellar structure in retrograded tigernut starch gels, distinct from the characteristic lamellar peak of native starch. The semi-crystalline lamellar long period (*d*_Bragg_) is calculated using Bragg’s formula (*d* = 2π/*q*_max_), and the derived results are summarized in Table 2. A smaller *d*_Bragg_ value corresponds to a thinner or more densely packed semi-crystalline lamellar structure. According to Guinier’s law, i.e., *I*_(*q*)_ = *I*_0_exp(−*Rg*^2^*q*^2^/3), the relationship between the slope *β* of the fitted curve (Figure 4C) and the radius of gyration (*R*_g_) is derived as $R_{g}=\sqrt{-3\beta }$. After performing linear fitting on the Guinier plot in the low *q* region (*q*^2^ < 0.15 nm^−2^) to obtain the slope of the fitted straight line, the radius of gyration (*R*_g_) of the sample can be calculated. The *R*_g_, defined as the root-mean-square distance from the center of mass of the scattering particle, reflects the compactness of the starch molecular chain structure, and a larger *R*_g_ values indicates a looser arrangement of starch molecular chains. As shown in Table 2, during the retrogradation process, the *d*_Bragg_ shows a significant decreasing trend from 22.13 nm at 0 h to 18.55 nm (72 h) (*p* < 0.05), while the *R*_g_ of the retrograded tigernut starch gel presents a corresponding declining trend from 19.94 nm (0 h) to 15.93 nm (72 h) (*p* < 0.05). Combined with the complementary evidence from X-ray diffraction and FT-IR characterization in this study, these SAXS-derived parameters suggest a possible tendency toward tighter packing of rearranged double-helical chain segments within the dried gel matrix, rather than being interpreted as a direct, standalone structural proof from SAXS measurements alone. The concurrent decrease in *d*_Bragg_ and *R*_g_ values further indicates that retrogradation promotes the regular rearrangement of intra-lamellar double helices or enhances the packing density of the amorphous lamellar domains [35]. This reduction can be tentatively attributed to the retrogradation-induced contraction of rearranged double-helical chain segments, which reduces the size of starch molecular aggregates and thereby enhances the compactness of starch molecular chains.

#### 3.2.4. Fractal Structure

The relationship between the scattering vector and scattering intensity of the scatterer follows the power-law function: *I*_(*q*)_ = *I*_0_*q*^−α^, which can be further transformed to derive the double logarithmic curve ln *I*_(*q*)_ vs. ln *q* (Figure 4D) [20]. By fitting this curve in the low-*q* region, the obtained slope α can be used to calculate the fractal dimension, a key parameter describing the complexity and self-similarity of the sample’s scattering structure. The fractal dimension can be divided into two categories: surface fractal dimension (*D*_s_) and mass fractal dimension (*D*_m_) [36]. When −4 < *α* < −3, *D*_s_ = 6 + *α*, corresponding to a surface fractal that characterizes the surface smoothness of the scatterer. When *D*_s_ = 2, the scatterer exhibits an absolutely smooth surface, and a higher *D*_s_ value indicates a rougher sample surface. When −3 < α <−1, *D*_m_ = -α, corresponding to a mass fractal that serves as a direct indicator of material compactness. As summarized in Table 1, all retrograded samples exhibit mass fractal structures with *D*_m_ values ranging from 1.0026 to 1.5488, and *D*_m_ shows a significant increasing trend with the extension of retrogradation time. This result indicates that the rearrangement of microcrystalline structures during retrogradation enhances the overall compactness of the starch gel matrix, which is highly consistent with the decreasing trends of *R*_g_ and *d*_Bragg_ obtained in previous SAXS analyses.

### 3.3. Water Distribution in Dried Tigernut Starch Gels as Affected by Retrogradation

#### 3.3.1. Water Activity (a_w_)

The initial moisture content of the dried tigernut starch gel is a key variable that directly determines the puffing performance of starch matrix [10]. For starch gel samples with different retrogradation durations before drying, the final moisture content of all dried products is controlled at approximately 10% (Table 2), and no significant difference is observed among groups (9.85–10.02%, *p* > 0.05). The water activity (*a*_w_) of the samples is further measured, and the results are shown in Table 2. Within the retrogradation duration of 0–72 h, the *a*_w_ of all dried starch gels ranges from 0.3751 to 0.4032, reaching the peak value at 4 h of retrogradation and showing a gradual decreasing trend thereafter. According to Boischot et al. [37], *a*_w_ is a critical prerequisite indicator for starch puffing, which must fall within a specific reasonable range to avoid irreversible internal structural collapse during the puffing process. An appropriate glass transition temperature is conducive to the rapid phase transition of starch matrix and the sufficient accumulation of internal steam pressure, while *a*_w_ can significantly regulate the glass transition behavior of starch, thereby further altering the final puffing performance of the material [38].

#### 3.3.2. Moisture Distribution

**Figure 5:**
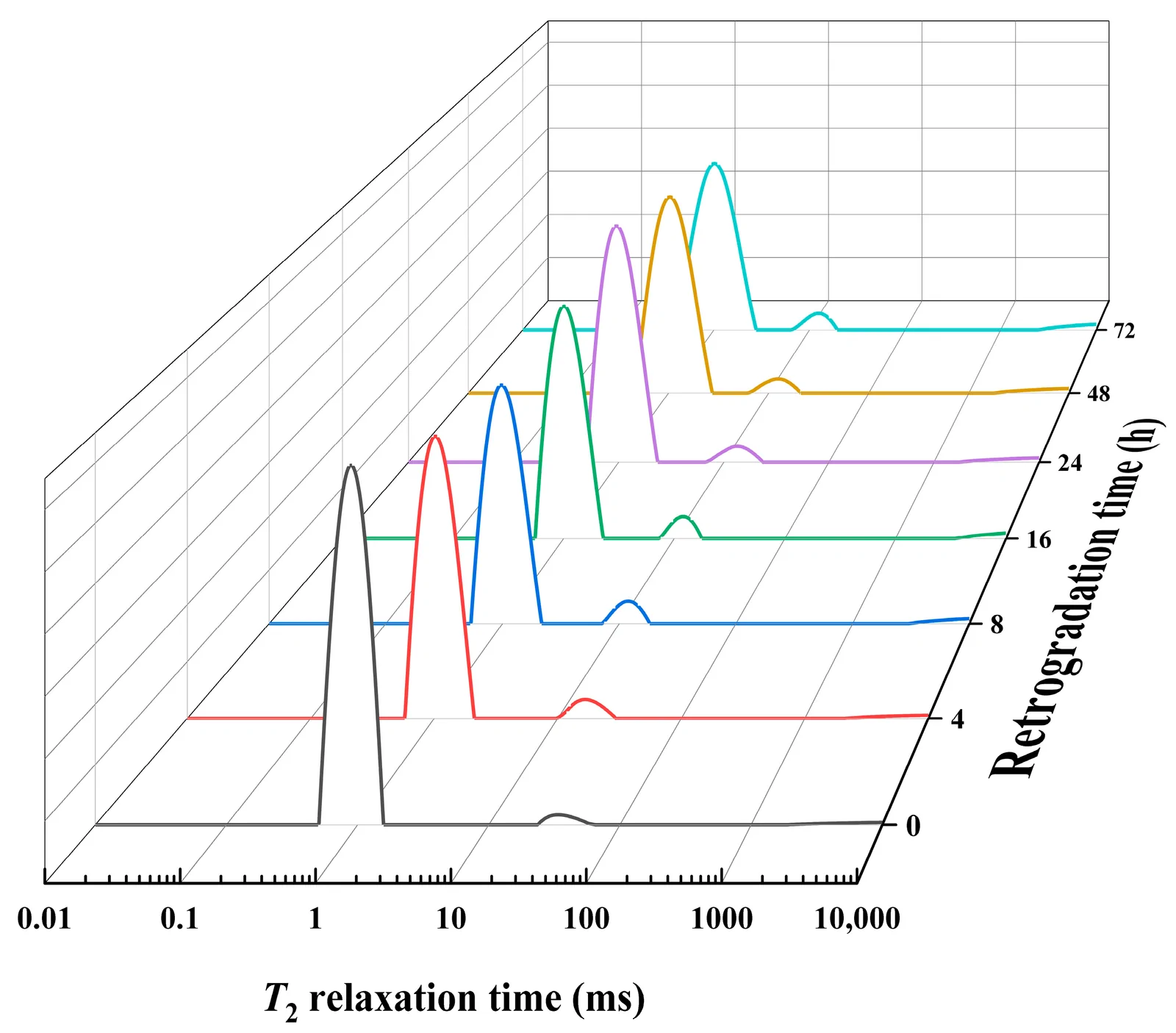
Water distribution in dried tigernut starch gels characterized by LF-NMR as a function of retrogradation time (0–72 h).

Figure 5 depicts the water distribution of dried tigernut starch gels before puffing as affected by different retrogradation durations. According to the transverse relaxation peak positions, the relaxation time *T*_21_ of all samples falls in the range of 0.94–1.04 ms, which corresponds to the tightly bound water fraction in the dried tigernut starch gel. The *T*_22_ of immobilized water ranges from 11.67 ms to 36.53 ms, while *T*_23_ (the indicator of free water) is defined as the relaxation component with *T*_2_ >1000 ms [39]. The bound water peak dominates all spectra, while the immobilized water peak is relatively weak. No distinct *T*_23_ relaxation peak (*T*_2_ > 1000 ms) was detected in all dried retrograded tigernut starch gel samples, confirming the absence of free water in these systems, which matches the low-moisture feature of the dried starch gel matrix [40]. In this context, only the corresponding peak area proportion *A*_23_ of the trace free water fraction below the detection threshold was recorded, instead of a complete measurable *T*_23_ value. A comparable observation has been documented in previous LF-NMR studies on dried corn resistant starch samples, where no detectable free water population was observed across all tested sample groups. [40]. As listed in Table 2, the relaxation time (*T*_21_) of bound water shows no significant regular trend among groups (*p* > 0.05), while the *T*_22_ of all retrograded samples is significantly lower than that of the 0 h sample (*p* < 0.05). This result indicates that the mobility of weakly bound water is reduced, and the immobilized water in the system is more tightly bound to the starch molecular chains. Additionally, with the extension of retrogradation time, the proportion of bound water *A*_21_ shows a significant decreasing trend, while the proportion of immobilized water *A*_22_ first increases and then decreases from 0 h to 72 h. The immobilized water fraction (*A*_22_) reaches its numerical peak at 8 h, and the values at 4 h and 16 h are statistically comparable to the peak value (*p* > 0.05). During retrogradation, starch molecules transform from a fully amorphous state to ordered double-helical structures and gradually form crystalline regions. The enhanced intermolecular interaction between starch chains expels the water molecules originally bound to starch via hydrogen bonds, thereby reducing the proportion of tightly bound water and increasing the proportion of immobilized water. However, among the discrete retrogradation durations tested in this study, samples subjected to retrogradation for 16 h and longer exhibited continuous recrystallization that further enhanced intermolecular interactions of starch molecules, accompanied by a measured decrease in the proportion of immobilized water. The free water fraction (*A*_23_) shows an overall gradual increasing trend throughout the retrogradation process as starch transitions from a disordered to an ordered molecular structure, with non-significant minor fluctuations at 16 h and 72 h (*p* > 0.05).

### 3.4. Thermal Puffing Properties and Textural Quality of Puffed Products

#### 3.4.1. Expansion Ratio and Specific Volume

Table 3 illustrates the effect of retrogradation duration on the physical properties of puffed tigernut starch gels. The expansion ratio initially increased and subsequently decreased with prolonged retrogradation time, reaching a peak value of 9.75 at 4 h, which was 30.70% significantly higher than that of the non-retrograded control (7.46) (*p* < 0.05). In contrast, after 72 h of retrogradation, the expansion ratio dropped to its minimum of 5.72, representing a 23.32% significant reduction compared to the control (*p* < 0.05). A similar trend was observed for specific volume (Table 3), which peaked at 8.42 mL/g at 4 h of retrogradation before gradually declining upon further storage, indicating a strong correlation between the two puffing quality parameters. These results, derived from the seven discrete retrogradation durations tested in this study, demonstrate that the 4 h retrograded sample achieved the greatest expansion ratio, while samples subjected to retrogradation for 16 h and longer showed inferior expansion compared to the non-retrograded control. The underlying mechanism can be attributed to the structural evolution induced by retrogradation. Short-term retrogradation facilitates the transformation of starch molecules from a disordered state into an ordered, yet still disruptible, lamellar framework. This moderately ordered structure favors effective encapsulation of immobilized water, as supported by the highest *A*_22_ peak value at 4 h observed in the LF-NMR measurements among all tested retrogradation groups. This state further facilitates rapid steam pressure generation during heating, which corresponds to the maximum expansion ratio recorded in this puffing experiment. [8]. Conversely, prolonged retrogradation fosters excessive molecular ordering and crystallinity, which elevates the energy barrier required for disrupting the internal structure. This impedes timely starch phase transition and steam pressure generation, ultimately compromising the completeness and extent of puffing.

#### 3.4.2. Textural Quality

Table 3 presents the textural properties of puffed tigernut starch products as a function of retrogradation degree. The hardness and crispness of the puffed samples vary significantly (*p* < 0.05) with different retrogradation durations. Notably, the sample subjected to 4 h of retrogradation demonstrates the most favorable textural profile, characterized by the minimum hardness (1165.53 g) and the maximum crispness (36.67). These values represent a significant 14.01% reduction in hardness and a 62.98% increase in crispness compared to the non-retrograded control (1355.43 g and 22.50, respectively) (*p* < 0.05). Extending retrogradation beyond 4 h leads to a progressive deterioration in textural quality, marked by a gradual increase in hardness and a concurrent decrease in crispness. The poorest textural attributes are observed after 72 h of retrogradation, with hardness and crispness reaching 2150.39 g and 16.50, respectively. The observed puffing behavior can be tentatively attributed to the formation of porous structure during thermal processing: rapid vaporization of internal moisture in the retrograded starch gel drives starch molecular rearrangement, and the expansion and subsequent stabilization of internal vapor chambers ultimately shape the final porous network. Consequently, the textural properties of the final puffed product are intrinsically linked to its pore architecture. Hardness is governed by the mechanical strength and thickness of the pore walls, where thinner walls facilitate easier fracture. Crispness, on the other hand, is positively correlated with the total number and uniformity of pores within the matrix, as a higher pore density typically enhances brittleness and fracturability [26]. The observed textural improvement at 4 h of retrogradation indicates that short-term molecular reorganization promotes the formation of a more uniform and favorable pore structure among the tested conditions, whereas prolonged retrogradation leads to excessive structural consolidation that impedes effective puffing and results in a denser, harder texture.

### 3.5. Correlation Analysis and Mechanistic Elucidation

**Figure 6:**
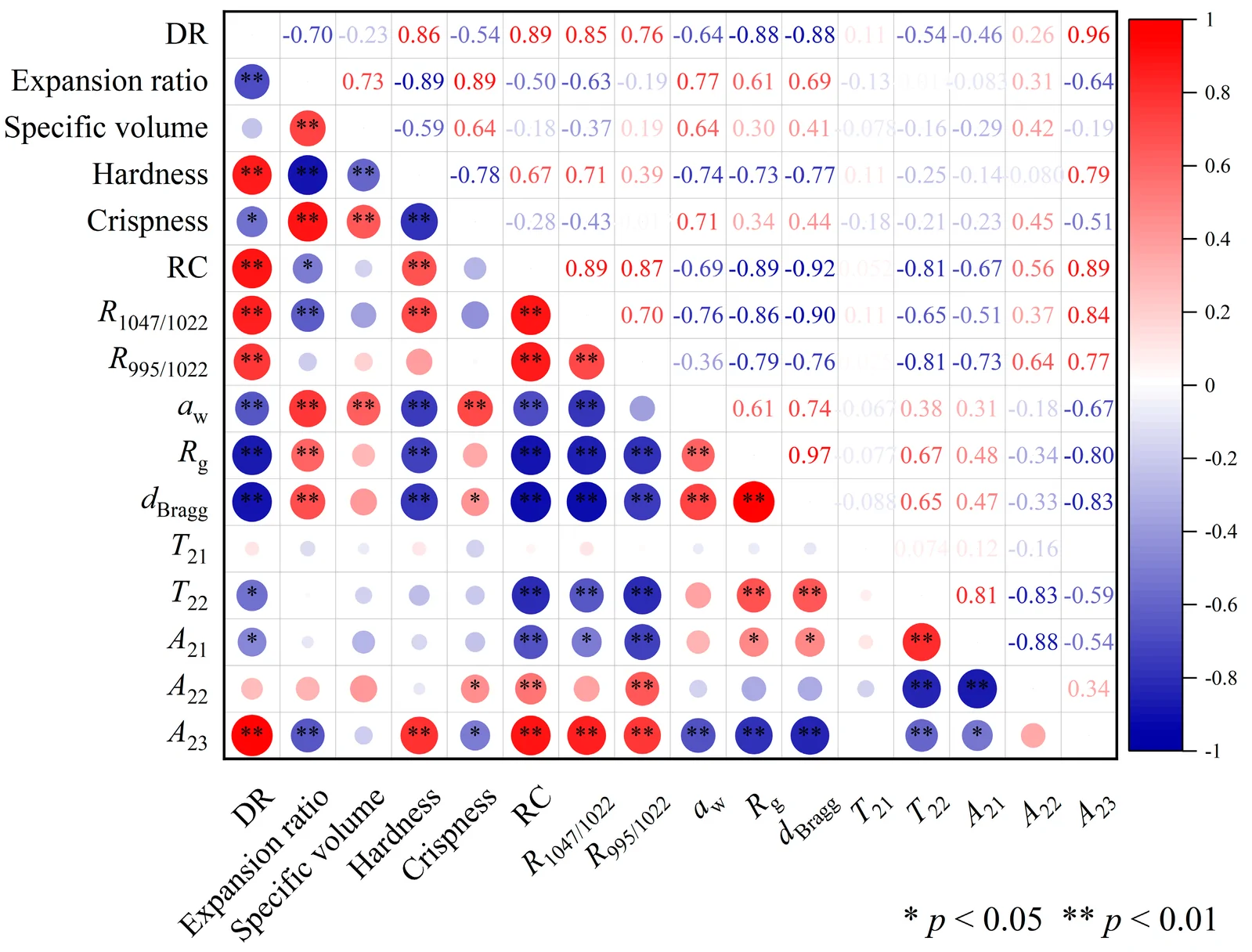
Pearson correlation analysis among multi-scale structural parameters, water distribution properties and puffing performance of retrograded tigernut starch gels.

To decipher the regulatory mechanism, Pearson correlation analysis was performed among key parameters, including puffing properties (expansion ratio, specific volume, hardness, and crispness), retrogradation degree, starch multi-scale structural indicators (*d*_Bragg_, *R*_g_, RC, and *R*_1047/1022_), and water state parameters (*a*_w_, *T*_21_, *T*_22_, *A*_21_, *A*_22_, and *A*_23_). Pearson correlation analysis was performed based on the seven retrogradation time points, with an effective sample size of *n* = 7 for each pairwise comparison. All correlations in this work are interpreted as exploratory statistical associations, and no causal relationship is implied. The correlation matrix (Figure 6) reveals that water activity (*a*_w_) exhibits an extremely significant correlation (*p* < 0.01) with all puffing quality parameters, establishing it as the dominant factor governing thermal puffing behavior. In specific, expansion ratio shows a significant positive correlation with *a*_w_, *R*_g_ and *d*_Bragg_ (*p* < 0.01), and a negative correlation with retrogradation degree, RC, *R*_1047/1022_, and *A*_23_ (*p* < 0.05). For textural properties, hardness is negatively correlated with *a*_w_, *R*_g_ and *d*_Bragg_ (*p* < 0.01), and positively correlated with retrogradation degree, RC, *R*_1047/1022_, and *A*_23_ (*p* < 0.01). Crispness is significantly negatively correlated with retrogradation degree and *A*_23_ (*p* < 0.05), while it is significantly positively correlated with *a*_w_ (*p* < 0.01), *d*_Bragg_ (*p* < 0.05) and immobilized water content *A*_22_ (*p* < 0.05). Furthermore, retrogradation degree shows extremely significant correlations (*p* < 0.01) with all structural parameters and significant correlations with *a*_w_ and several water distribution parameters (*T*_21_, *A*_21_, and *A*_23_).

This integrated analysis outlines a data-consistent plausible mechanism that aligns well with all measured parameters in this study: Variation in retrogradation duration drives multi-scale structural evolution of tigernut starch matrices (decreased *d*_Bragg_ and *R*_g_, increased relative crystallinity and short-range order *R*_1047/1022_), which further modulates water distribution and availability reflected by the measured water activity (*a*_w_), immobilized water fraction *A*_22_ and bound water fraction *A*_23_, and ultimately correlates with the observed thermal puffing performance and final product texture. Among the seven discrete retrogradation time points tested in this work, the 4 h retrograded sample formed a moderate level of molecular order, creating a moderately ordered lamellar framework that retained the highest content of immobilized water. This measured state corresponded to the highest *a*_w_ value recorded in the experiment, which was highly likely to provide a favorable precondition for sufficient steam generation during thermal puffing, and matched the highest expansion ratio and the most favorable crispy texture observed in this study. Conversely, samples subjected to retrogradation for 16 h and longer developed excessive molecular ordering and crystallinity, which was accompanied by a reduced *a*_w_ value and a more rigid starch matrix that restricted structural disruption during heating, and this observed trend correlates with the impaired puffing performance and harder, less crisp final product recorded in our tests.

## 4. Conclusions

This study investigated the impact of retrogradation time (0–72 h at 4 °C) on the thermal puffing performance of tigernut starch. The results confirm that retrogradation time acts as a critical governing factor for the multi-scale structural evolution of dried starch gels. Prolonged refrigeration storage significantly increased the degree of retrogradation, driving the transformation of starch molecular architecture towards a more ordered and compact state. This structural trend is supported by the synchronous enhancement of relative crystallinity and short-range molecular order, accompanied by the reduction in the radius of gyration and lamellar long period, which collectively indicate a tighter molecular packing configuration. Among all seven tested retrogradation time points, the sample subjected to 4 h of retrogradation presented the best comprehensive thermal puffing performance. This “moderate retrogradation” phenomenon can be attributed to the formation of a moderately ordered network structure. Among all tested groups, this state corresponds to the highest content of immobilized water in the system, which further contributes to the most favorable internal vapor pressure condition for puffing. In contrast, 16 h or longer retrogradation induces over-compacted crystalline structures that restrict the expansion of starch matrix during heating. Correlation analysis identifies water activity as the dominant factor controlling puffing behavior, while the crispness property of puffed products is co-regulated by water activity and the nanoscale lamellar structural feature. These findings establish retrogradation time as a key tunable parameter for regulating the interconnection among multi-scale structure, water distribution and puffing functionality in tigernut starch systems. This work offers a targeted structural regulation strategy for the manufacturing of high-quality indirectly thermally starchy puffed foods.

## Figures and Tables

**Table 1:** Relative crystallinity, short-range ordered degree and lamellar structure parameters of dried retrograded tigernut starch gels as a function of retrogradation time (0–72 h).

Retrogradation Time/h	*R*_1047/1022_	*R*_995/1022_	RC (%)	*d*_Bragg_ (nm)	*R*_g_ (nm)	*D*_m_
0	0.5086 ± 0.004 ^a^	1.2368 ± 0.004 ^a^	5.44 ± 0.06 ^a^	22.13 ± 0.18 ^d^	19.94 ± 0.002 ^g^	1.55 ± 0.01 ^f^
4	0.5260 ± 0.009 ^a^	1.3239 ± 0.003 ^b^	11.09 ± 1.08 ^b^	20.80 ± 0.47 ^c^	18.93 ± 0.003 ^f^	1.42 ± 0.02 ^d^
8	0.5628 ± 0.004 ^b^	1.3244 ± 0.019 ^b^	13.86 ± 0.31 ^c^	21.11 ± 0.16 ^c^	18.41 ± 0.000 ^e^	1.46 ± 0.01 ^e^
16	0.5738 ± 0.011 ^b^	1.3264 ± 0.031 ^b^	14.36 ± 0.84 ^c^	19.33 ± 0.14 ^b^	16.82 ± 0.002 ^c^	1.09 ± 0.01 ^b^
24	0.5898 ± 0.013 ^bc^	1.3612 ± 0.015 ^c^	16.53 ± 0.49 ^d^	18.55 ± 0.13 ^a^	16.17 ± 0.004 ^b^	1.01 ± 0.01 ^a^
48	0.5854 ± 0.030 ^bc^	1.3583 ± 0.004 ^c^	16.50 ± 0.54 ^d^	19.33 ± 0.14 ^b^	17.08 ± 0.001 ^d^	1.22 ± 0.02 ^c^
72	0.6039 ± 0.016 ^c^	1.3383 ± 0.009 ^bc^	17.99 ± 0.39 ^e^	18.80 ± 0.13 ^a^	15.93 ± 0.004 ^a^	1.00 ± 0.01 ^a^

Notes: RC, relative crystallinity; *R*_1047/1022_, band intensity ratio of the deconvoluted FTIR spectrum at 1047 cm^−1^ and 1022 cm^−1^; *R*_995/1022_, band intensity ratio of the deconvoluted FTIR spectrum at 995 cm^−1^ and 1022 cm^−1^; *d*_Bragg_, Bragg long period; *R*_g_, radius of gyration; *D*_m_, mass fractal dimension. Values in the same column followed by different lowercase letters are significantly different (*p* < 0.05).

**Table 2:** The water content, water activity (*a*_w_), and water distribution parameters of dried retrograded tigernut starch gels at different retrogradation times (0–72 h).

Retrogradation Time/h	Water Content (g/100 g)	*a*_w_	*T*_21_ (ms)	*T*_22_ (ms)	*A*_21_ (%)	*A*_22_ (%)	*A*_23_ (%)
0	9.85 ± 0.17 ^a^	0.3967 ± 0.002 ^e^	0.98 ± 0.10 ^a^	36.53 ± 2.52 ^b^	96.85 ± 0.24 ^c^	2.19 ± 0.10 ^a^	0.95 ± 0.17 ^a^
4	9.98 ± 0.04 ^a^	0.4032 ± 0.002 ^f^	1.02 ± 0.01 ^a^	13.05 ± 3.52 ^a^	93.20 ± 0.43 ^b^	5.61 ± 0.51 ^de^	1.19 ± 0.08 ^b^
8	9.98 ± 0.02 ^a^	0.3889 ± 0.001 ^bcd^	0.94 ± 0.03 ^a^	11.89 ± 0.49 ^a^	92.50 ± 0.53 ^ab^	5.96 ± 0.49 ^e^	1.55 ± 0.07 ^c^
16	9.80 ± 0.33 ^a^	0.3851 ± 0.002 ^b^	0.99 ± 0.04 ^a^	11.95 ± 0.66 ^a^	93.72 ± 1.26 ^b^	5.53 ± 0.00 ^cde^	1.45 ± 0.11 ^c^
24	9.98 ± 0.09 ^a^	0.3918 ± 0.002 ^cd^	0.96 ± 0.01 ^a^	13.28 ± 0.18 ^a^	93.48 ± 0.17 ^b^	4.59 ± 0.23 ^b^	1.93 ± 0.05 ^d^
48	10.02 ± 0.07 ^a^	0.3873 ± 0.001 ^bc^	0.98 ± 0.11 ^a^	11.67 ± 0.73 ^a^	93.04 ± 0.88 ^ab^	4.74 ± 0.87 ^bc^	2.22 ± 0.05 ^e^
72	10.02 ± 0.08 ^a^	0.3751 ± 0.002 ^a^	1.04 ± 0.03 ^a^	12.06 ± 0.33 ^a^	91.92 ± 0.49 ^a^	4.92 ± 0.36 ^bcd^	2.11 ± 0.12 ^e^

Notes: no distinct *T*_23_ relaxation peak (*T*_2_ > 1000 ms) was detected. *T*_21_ and *T*_22_ are the relaxation times of bound water and immobilized water, respectively, while *A*_21_, *A*_22_, and *A*_23_ are the corresponding proportions of bound water, immobilized water, and free water, respectively. Values in the same column followed by different lowercase letters are significantly different (*p* < 0.05).

**Table 3:** Thermal puffing properties and textural parameters of puffed products from dried retrograded tigernut starch gels as a function of retrogradation time (0–72 h).

Retrogradation Time/h	Sample	Expansion Ratio	Specific Volume/(mL/g)	Hardness/g	Crispness/Numbers
0	TS-0h	7.46 ± 0.31 ^c^	7.25 ± 0.33 ^ab^	1355.43 ± 128.06 ^bc^	22.50 ± 0.50 ^b^
4	TS-4h	9.75 ± 0.05 ^e^	8.42 ± 0.37 ^d^	1165.53 ± 75.45 ^a^	36.67 ± 2.08 ^e^
8	TS-8h	8.55 ± 0.25 ^d^	7.87 ± 0.14 ^cd^	1209.65 ± 100.04 ^ab^	30.33 ± 2.31 ^d^
16	TS-16h	7.38 ± 0.29 ^c^	7.17 ± 0.59 ^ab^	1438.93 ± 80.90 ^c^	27.00 ± 1.00 ^c^
24	TS-24h	6.75 ± 0.17 ^b^	7.49 ± 0.03 ^bc^	1742.65 ± 61.39 ^d^	26.33 ± 0.58 ^c^
48	TS-48h	6.83 ± 0.04 ^b^	7.96 ± 0.04 ^cd^	1783.05 ± 81.86 ^d^	21.00 ± 2.00 ^b^
72	TS-72h	5.72 ± 0.29 ^a^	6.67 ± 0.34 ^a^	2150.39 ± 29.88 ^e^	16.50 ± 2.50 ^a^

Notes: Values in the same column followed by different lowercase letters (a–e) indicate significant differences at *p* < 0.05.

## Data Availability

The original contributions presented in this study are included in the article. Further inquiries can be directed to the corresponding author.
